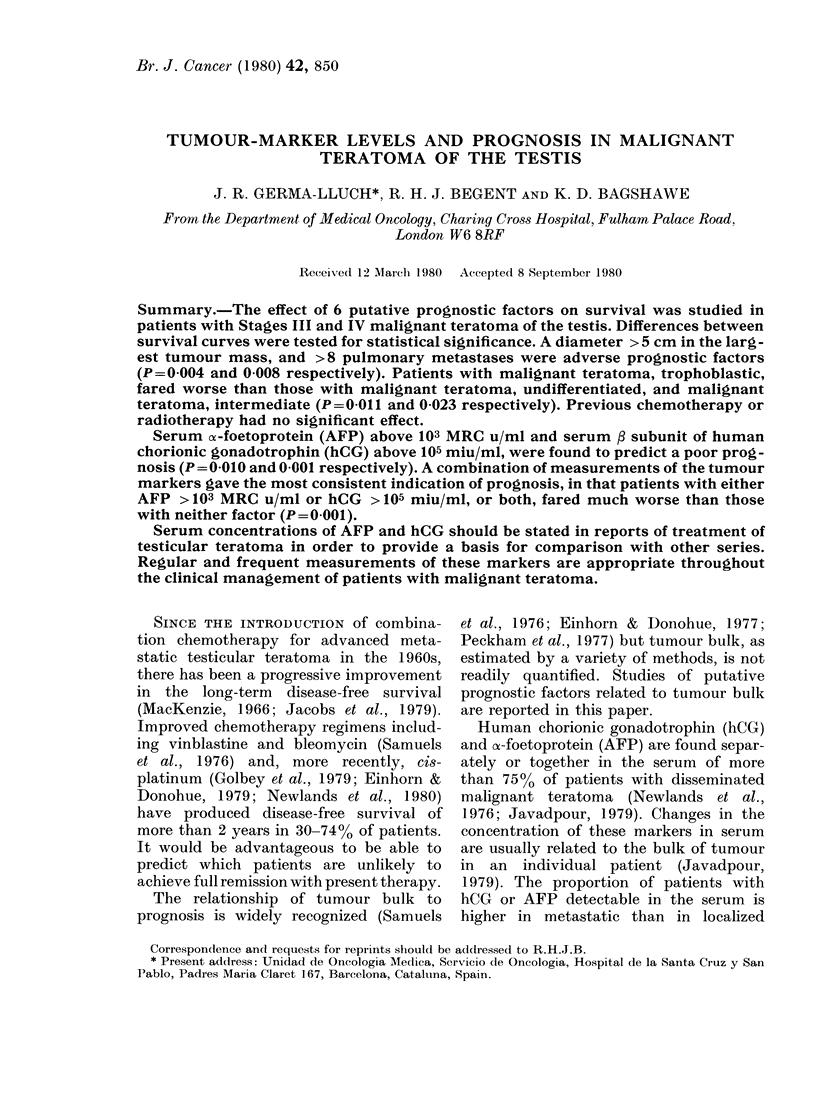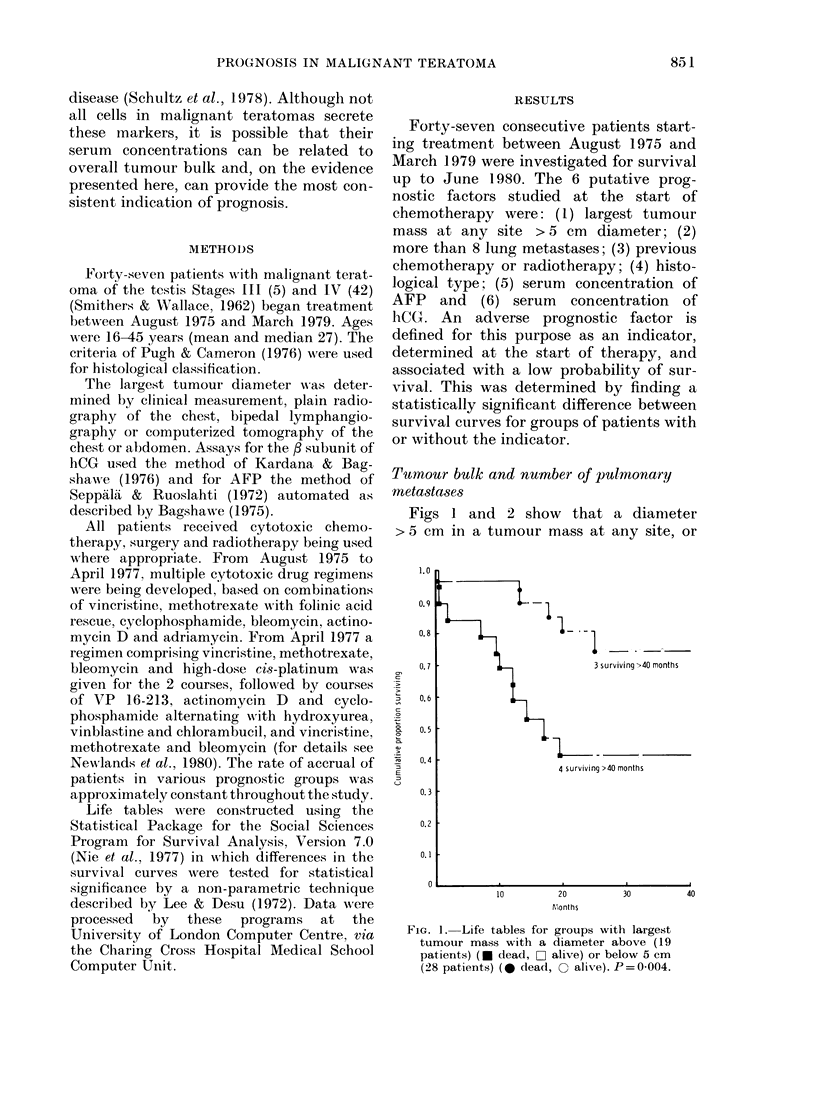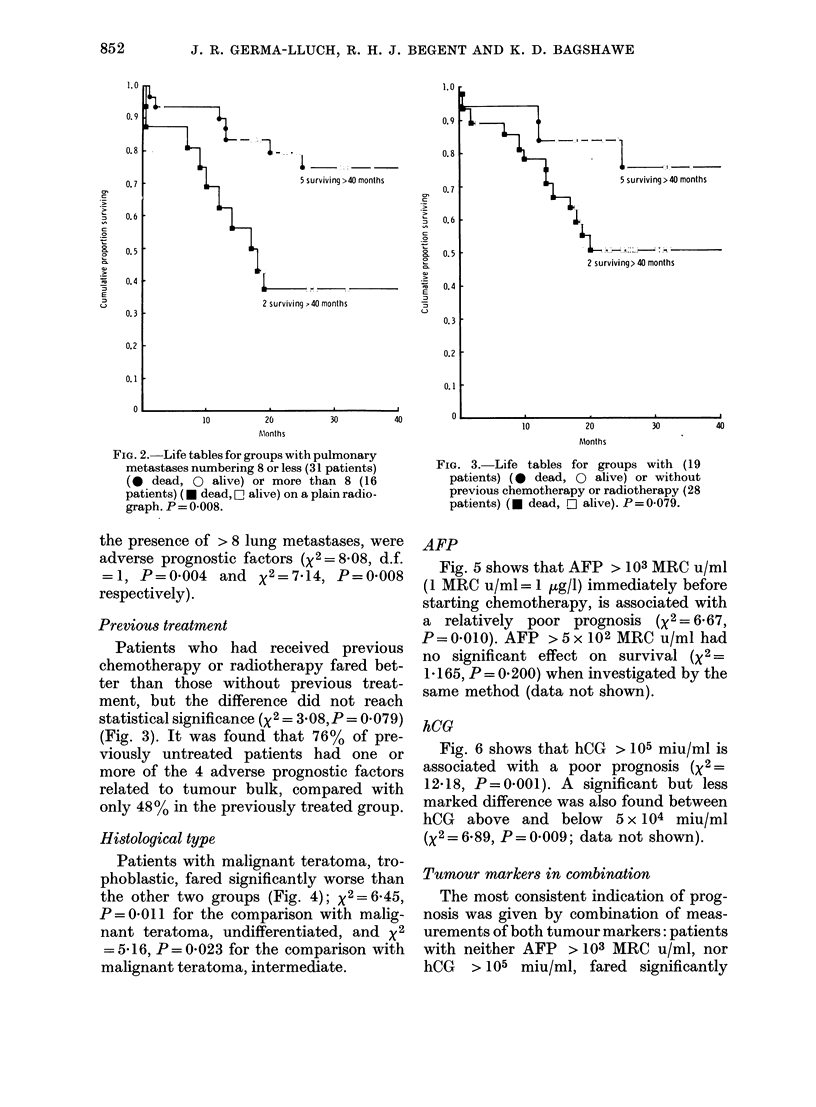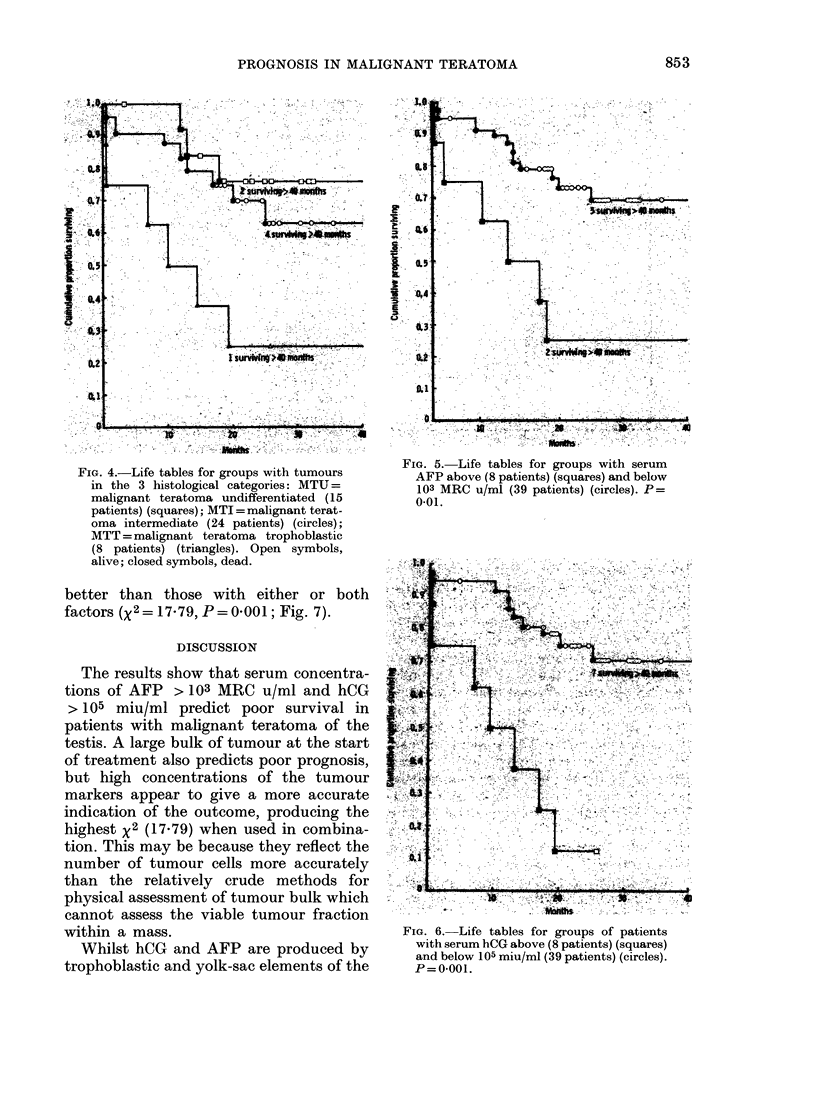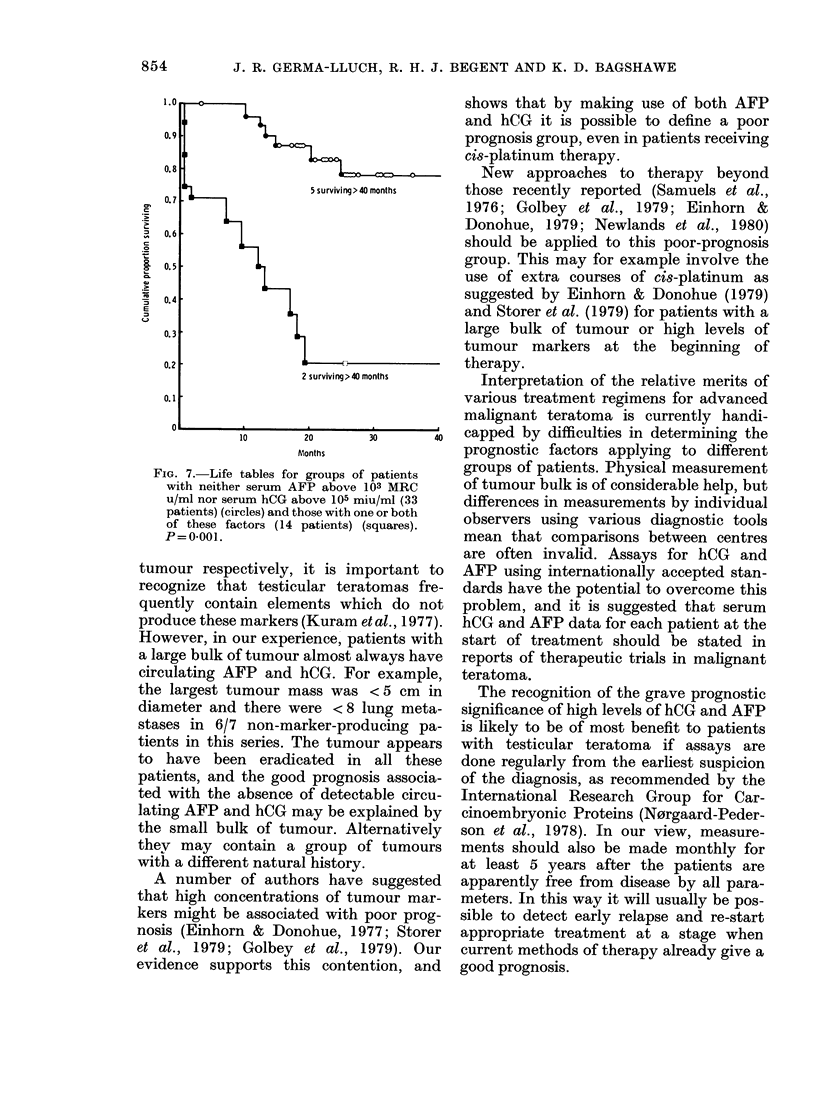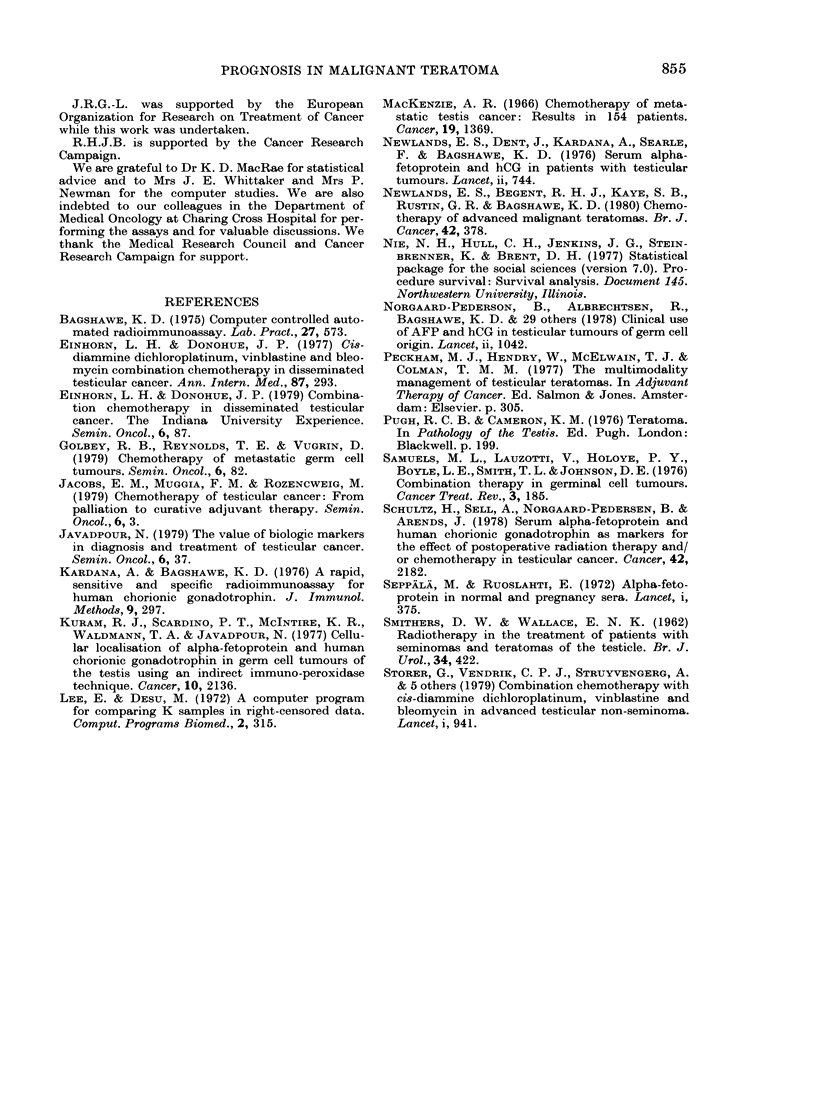# Tumour-marker levels and prognosis in malignant teratoma of the testis.

**DOI:** 10.1038/bjc.1980.332

**Published:** 1980-12

**Authors:** J. R. Germa-Lluch, R. H. Begent, K. D. Bagshawe

## Abstract

The effect of 6 putative prognostic factors on survival was studied in patients with Stages III and IV malignant teratoma of the testis. Differences between survival curves were tested for statistical significance. A diameter greater than 5 cm in the largest tumour mass, and greater than 8 pulmonary metastases were adverse prognostic factors (P = 0.004 and 0.008 respectively). Patients with malignant teratoma, trophoblastic, fared worse than those with malignant teratoma, undifferentiated, and malignant teratoma, intermediate (P = 0.011 and 0.023 respectively). Previous chemotherapy or radiotherapy had no significant effect. Serum alpha-foetoprotein (AFP) above 10(3) MRC u/ml and serum beta subunit of human chorionic gonadotrophin (hCG) above 10(5) miu/ml, were found to predict a poor prognosis (P = 0.010 and 0.001 respectively). A combination of measurements of the tumour markers gave the most consistent indication of prognosis, in that patients with either AFP greater than 10(3) MRC u/ml or hCG greater than 10(5) miu/ml, or both, fared much worse than those with neither factor (P = 0.001). Serum concentrations of AFP and hCG should be stated in reports of treatment of testicular teratoma in order to provide a basis for comparison with other series. Regular and frequent measurements of these markers are appropriate throughout the clinical management of patients with malignant teratoma.


*Br. J. Cancer* (1980) **42**, 850

### TUMOUR-MARKER LEVELS AND PROGNOSIS IN MALIGNANT TERATOMA OF THE TESTIS

J. R. GERMA-LLUCH*, R. H. J. BEGENT AND K. D. BAGSHAWE

*From the Department of Medical Oncology, Charing Cross Hospital, Fulham Palace Road, London W6 8RF*

Received 12 March 1980 Accepted 8 September 1980

**Summary.—The effect of 6 putative prognostic factors on survival was studied in patients with Stages III and IV malignant teratoma of the testis. Differences between survival curves were tested for statistical significance. A diameter >5 cm in the largest tumour mass, and >8 pulmonary metastases were adverse prognostic factors ($P=0{\cdot}004$ and 0·008 respectively). Patients with malignant teratoma, trophoblastic, fared worse than those with malignant teratoma, undifferentiated, and malignant teratoma, intermediate ($P=0{\cdot}011$ and 0·023 respectively). Previous chemotherapy or radiotherapy had no significant effect.**

**Serum α-foetoprotein (AFP) above $10^3$ MRC u/ml and serum β subunit of human chorionic gonadotrophin (hCG) above $10^5$ miu/ml, were found to predict a poor prognosis ($P=0{\cdot}010$ and 0·001 respectively). A combination of measurements of the tumour markers gave the most consistent indication of prognosis, in that patients with either AFP $>10^3$ MRC u/ml or hCG $>10^5$ miu/ml, or both, fared much worse than those with neither factor ($P=0{\cdot}001$).**

**Serum concentrations of AFP and hCG should be stated in reports of treatment of testicular teratoma in order to provide a basis for comparison with other series. Regular and frequent measurements of these markers are appropriate throughout the clinical management of patients with malignant teratoma.**

SINCE THE INTRODUCTION of combination chemotherapy for advanced metastatic testicular teratoma in the 1960s, there has been a progressive improvement in the long-term disease-free survival (MacKenzie, 1966; Jacobs *et al.*, 1979). Improved chemotherapy regimens including vinblastine and bleomycin (Samuels *et al.*, 1976) and, more recently, *cis*-platinum (Golbey *et al.*, 1979; Einhorn & Donohue, 1979; Newlands *et al.*, 1980) have produced disease-free survival of more than 2 years in 30–74% of patients. It would be advantageous to be able to predict which patients are unlikely to achieve full remission with present therapy.

The relationship of tumour bulk to prognosis is widely recognized (Samuels *et al.*, 1976; Einhorn & Donohue, 1977; Peckham *et al.*, 1977) but tumour bulk, as estimated by a variety of methods, is not readily quantified. Studies of putative prognostic factors related to tumour bulk are reported in this paper.

Human chorionic gonadotrophin (hCG) and α-foetoprotein (AFP) are found separately or together in the serum of more than 75% of patients with disseminated malignant teratoma (Newlands *et al.*, 1976; Javadpour, 1979). Changes in the concentration of these markers in serum are usually related to the bulk of tumour in an individual patient (Javadpour, 1979). The proportion of patients with hCG or AFP detectable in the serum is higher in metastatic than in localized

Correspondence and requests for reprints should be addressed to R.H.J.B.

* Present address: Unidad de Oncologia Medica, Servicio de Oncologia, Hospital de la Santa Cruz y San Pablo, Padres Maria Claret 167, Barcelona, Cataluna, Spain.

disease (Schultz *et al.*, 1978). Although not all cells in malignant teratomas secrete these markers, it is possible that their serum concentrations can be related to overall tumour bulk and, on the evidence presented here, can provide the most consistent indication of prognosis.

### METHODS

Forty-seven patients with malignant teratoma of the testis Stages III (5) and IV (42) (Smithers & Wallace, 1962) began treatment between August 1975 and March 1979. Ages were 16–45 years (mean and median 27). The criteria of Pugh & Cameron (1976) were used for histological classification.

The largest tumour diameter was determined by clinical measurement, plain radiography of the chest, bipedal lymphangiography or computerized tomography of the chest or abdomen. Assays for the $\beta$ subunit of hCG used the method of Kardana & Bagshawe (1976) and for AFP the method of Seppälä & Ruoslahti (1972) automated as described by Bagshawe (1975).

All patients received cytotoxic chemotherapy, surgery and radiotherapy being used where appropriate. From August 1975 to April 1977, multiple cytotoxic drug regimens were being developed, based on combinations of vincristine, methotrexate with folinic acid rescue, cyclophosphamide, bleomycin, actinomycin D and adriamycin. From April 1977 a regimen comprising vincristine, methotrexate, bleomycin and high-dose *cis*-platinum was given for the 2 courses, followed by courses of VP 16-213, actinomycin D and cyclophosphamide alternating with hydroxyurea, vinblastine and chlorambucil, and vincristine, methotrexate and bleomycin (for details see Newlands *et al.*, 1980). The rate of accrual of patients in various prognostic groups was approximately constant throughout the study.

Life tables were constructed using the Statistical Package for the Social Sciences Program for Survival Analysis, Version 7.0 (Nie *et al.*, 1977) in which differences in the survival curves were tested for statistical significance by a non-parametric technique described by Lee & Desu (1972). Data were processed by these programs at the University of London Computer Centre, *via* the Charing Cross Hospital Medical School Computer Unit.

### RESULTS

Forty-seven consecutive patients starting treatment between August 1975 and March 1979 were investigated for survival up to June 1980. The 6 putative prognostic factors studied at the start of chemotherapy were: (1) largest tumour mass at any site $>5$ cm diameter; (2) more than 8 lung metastases; (3) previous chemotherapy or radiotherapy; (4) histological type; (5) serum concentration of AFP and (6) serum concentration of hCG. An adverse prognostic factor is defined for this purpose as an indicator, determined at the start of therapy, and associated with a low probability of survival. This was determined by finding a statistically significant difference between survival curves for groups of patients with or without the indicator.

### *Tumour bulk and number of pulmonary metastases*

Figs 1 and 2 show that a diameter $>5$ cm in a tumour mass at any site, or

FIG. 1.—Life tables for groups with largest tumour mass with a diameter above (19 patients) (■ dead, □ alive) or below 5 cm (28 patients) (● dead, ○ alive). $P=0{\cdot}004$.

FIG. 2.—Life tables for groups with pulmonary metastases numbering 8 or less (31 patients) (● dead, ○ alive) or more than 8 (16 patients) (■ dead, □ alive) on a plain radiograph. $P=0\cdot008$.

FIG. 3.—Life tables for groups with (19 patients) (● dead, ○ alive) or without previous chemotherapy or radiotherapy (28 patients) (■ dead, □ alive). $P=0\cdot079$.

the presence of $>8$ lung metastases, were adverse prognostic factors ($\chi^2=8\cdot08$, d.f. $=1$, $P=0\cdot004$ and $\chi^2=7\cdot14$, $P=0\cdot008$ respectively).

*Previous treatment*

Patients who had received previous chemotherapy or radiotherapy fared better than those without previous treatment, but the difference did not reach statistical significance ($\chi^2=3\cdot08, P=0\cdot079$) (Fig. 3). It was found that 76% of previously untreated patients had one or more of the 4 adverse prognostic factors related to tumour bulk, compared with only 48% in the previously treated group.

*Histological type*

Patients with malignant teratoma, trophoblastic, fared significantly worse than the other two groups (Fig. 4); $\chi^2=6\cdot45$, $P=0\cdot011$ for the comparison with malignant teratoma, undifferentiated, and $\chi^2=5\cdot16$, $P=0\cdot023$ for the comparison with malignant teratoma, intermediate.

*AFP*

Fig. 5 shows that AFP $>10^3$ MRC u/ml (1 MRC u/ml $=1$ $\mu$g/l) immediately before starting chemotherapy, is associated with a relatively poor prognosis ($\chi^2=6\cdot67$, $P=0\cdot010$). AFP $>5\times10^2$ MRC u/ml had no significant effect on survival ($\chi^2=1\cdot165$, $P=0\cdot200$) when investigated by the same method (data not shown).

*hCG*

Fig. 6 shows that hCG $>10^5$ miu/ml is associated with a poor prognosis ($\chi^2=12\cdot18$, $P=0\cdot001$). A significant but less marked difference was also found between hCG above and below $5\times10^4$ miu/ml ($\chi^2=6\cdot89$, $P=0\cdot009$; data not shown).

*Tumour markers in combination*

The most consistent indication of prognosis was given by combination of measurements of both tumour markers: patients with neither AFP $>10^3$ MRC u/ml, nor hCG $>10^5$ miu/ml, fared significantly

FIG. 4.—Life tables for groups with tumours in the 3 histological categories: MTU = malignant teratoma undifferentiated (15 patients) (squares); MTI = malignant teratoma intermediate (24 patients) (circles); MTT = malignant teratoma trophoblastic (8 patients) (triangles). Open symbols, alive; closed symbols, dead.

better than those with either or both factors ($\chi^2 = 17 \cdot 79$, $P = 0 \cdot 001$; Fig. 7).

### DISCUSSION

The results show that serum concentrations of AFP $> 10^3$ MRC u/ml and hCG $> 10^5$ miu/ml predict poor survival in patients with malignant teratoma of the testis. A large bulk of tumour at the start of treatment also predicts poor prognosis, but high concentrations of the tumour markers appear to give a more accurate indication of the outcome, producing the highest $\chi^2$ (17·79) when used in combination. This may be because they reflect the number of tumour cells more accurately than the relatively crude methods for physical assessment of tumour bulk which cannot assess the viable tumour fraction within a mass.

Whilst hCG and AFP are produced by trophoblastic and yolk-sac elements of the

FIG. 5.—Life tables for groups with serum AFP above (8 patients) (squares) and below $10^3$ MRC u/ml (39 patients) (circles). $P = 0 \cdot 01$.

FIG. 6.—Life tables for groups of patients with serum hCG above (8 patients) (squares) and below $10^5$ miu/ml (39 patients) (circles). $P = 0 \cdot 001$.

FIG. 7.—Life tables for groups of patients with neither serum AFP above $10^3$ MRC u/ml nor serum hCG above $10^5$ miu/ml (33 patients) (circles) and those with one or both of these factors (14 patients) (squares). $P=0 \cdot 001$.

tumour respectively, it is important to recognize that testicular teratomas frequently contain elements which do not produce these markers (Kuram *et al.*, 1977). However, in our experience, patients with a large bulk of tumour almost always have circulating AFP and hCG. For example, the largest tumour mass was $<5$ cm in diameter and there were $<8$ lung metastases in 6/7 non-marker-producing patients in this series. The tumour appears to have been eradicated in all these patients, and the good prognosis associated with the absence of detectable circulating AFP and hCG may be explained by the small bulk of tumour. Alternatively they may contain a group of tumours with a different natural history.

A number of authors have suggested that high concentrations of tumour markers might be associated with poor prognosis (Einhorn & Donohue, 1977; Storer *et al.*, 1979; Golbey *et al.*, 1979). Our evidence supports this contention, and shows that by making use of both AFP and hCG it is possible to define a poor prognosis group, even in patients receiving *cis*-platinum therapy.

New approaches to therapy beyond those recently reported (Samuels *et al.*, 1976; Golbey *et al.*, 1979; Einhorn & Donohue, 1979; Newlands *et al.*, 1980) should be applied to this poor-prognosis group. This may for example involve the use of extra courses of *cis*-platinum as suggested by Einhorn & Donohue (1979) and Storer *et al.* (1979) for patients with a large bulk of tumour or high levels of tumour markers at the beginning of therapy.

Interpretation of the relative merits of various treatment regimens for advanced malignant teratoma is currently handicapped by difficulties in determining the prognostic factors applying to different groups of patients. Physical measurement of tumour bulk is of considerable help, but differences in measurements by individual observers using various diagnostic tools mean that comparisons between centres are often invalid. Assays for hCG and AFP using internationally accepted standards have the potential to overcome this problem, and it is suggested that serum hCG and AFP data for each patient at the start of treatment should be stated in reports of therapeutic trials in malignant teratoma.

The recognition of the grave prognostic significance of high levels of hCG and AFP is likely to be of most benefit to patients with testicular teratoma if assays are done regularly from the earliest suspicion of the diagnosis, as recommended by the International Research Group for Carcinoembryonic Proteins (Nørgaard-Pederson *et al.*, 1978). In our view, measurements should also be made monthly for at least 5 years after the patients are apparently free from disease by all parameters. In this way it will usually be possible to detect early relapse and re-start appropriate treatment at a stage when current methods of therapy already give a good prognosis.

J.R.G.-L. was supported by the European Organization for Research on Treatment of Cancer while this work was undertaken.

R.H.J.B. is supported by the Cancer Research Campaign.

We are grateful to Dr K. D. MacRae for statistical advice and to Mrs J. E. Whittaker and Mrs P. Newman for the computer studies. We are also indebted to our colleagues in the Department of Medical Oncology at Charing Cross Hospital for performing the assays and for valuable discussions. We thank the Medical Research Council and Cancer Research Campaign for support.

### REFERENCES

Bagshawe, K. D. (1975) Computer controlled automated radioimmunoassay. *Lab. Pract.*, **27**, 573.

Einhorn, L. H. & Donohue, J. P. (1977) *Cis*-diammine dichloroplatinum, vinblastine and bleomycin combination chemotherapy in disseminated testicular cancer. *Ann. Intern. Med.*, **87**, 293.

Einhorn, L. H. & Donohue, J. P. (1979) Combination chemotherapy in disseminated testicular cancer. The Indiana University Experience. *Semin. Oncol.*, **6**, 87.

Golbey, R. B., Reynolds, T. E. & Vugrin, D. (1979) Chemotherapy of metastatic germ cell tumours. *Semin. Oncol.*, **6**, 82.

Jacobs, E. M., Muggia, F. M. & Rozencweig, M. (1979) Chemotherapy of testicular cancer: From palliation to curative adjuvant therapy. *Semin. Oncol.*, **6**, 3.

Javadpour, N. (1979) The value of biologic markers in diagnosis and treatment of testicular cancer. *Semin. Oncol.*, **6**, 37.

Kardana, A. & Bagshawe, K. D. (1976) A rapid, sensitive and specific radioimmunoassay for human chorionic gonadotrophin. *J. Immunol. Methods*, **9**, 297.

Kuram, R. J., Scardino, P. T., McIntire, K. R., Waldmann, T. A. & Javadpour, N. (1977) Cellular localisation of alpha-fetoprotein and human chorionic gonadotrophin in germ cell tumours of the testis using an indirect immuno-peroxidase technique. *Cancer*, **10**, 2136.

Lee, E. & Desu, M. (1972) A computer program for comparing K samples in right-censored data. *Comput. Programs Biomed.*, **2**, 315.

MacKenzie, A. R. (1966) Chemotherapy of metastatic testis cancer: Results in 154 patients. *Cancer*, **19**, 1369.

Newlands, E. S., Dent, J., Kardana, A., Searle, F. & Bagshawe, K. D. (1976) Serum alpha-fetoprotein and hCG in patients with testicular tumours. *Lancet*, ii, 744.

Newlands, E. S., Begent, R. H. J., Kaye, S. B., Rustin, G. R. & Bagshawe, K. D. (1980) Chemotherapy of advanced malignant teratomas. *Br. J. Cancer*, **42**, 378.

Nie, N. H., Hull, C. H., Jenkins, J. G., Steinbrenner, K. & Brent, D. H. (1977) Statistical package for the social sciences (version 7.0). Procedure survival: Survival analysis. *Document 145. Northwestern University, Illinois.*

Norgaard-Pederson, B., Albrechtsen, R., Bagshawe, K. D. & 29 others (1978) Clinical use of AFP and hCG in testicular tumours of germ cell origin. *Lancet*, ii, 1042.

Peckham, M. J., Hendry, W., McElwain, T. J. & Colman, T. M. M. (1977) The multimodality management of testicular teratomas. In *Adjuvant Therapy of Cancer*. Ed. Salmon & Jones. Amsterdam: Elsevier. p. 305.

Pugh, R. C. B. & Cameron, K. M. (1976) Teratoma. In *Pathology of the Testis*. Ed. Pugh. London: Blackwell. p. 199.

Samuels, M. L., Lauzotti, V., Holoye, P. Y., Boyle, L. E., Smith, T. L. & Johnson, D. E. (1976) Combination therapy in germinal cell tumours. *Cancer Treat. Rev.*, **3**, 185.

Schultz, H., Sell, A., Norgaard-Pedersen, B. & Arends, J. (1978) Serum alpha-fetoprotein and human chorionic gonadotrophin as markers for the effect of postoperative radiation therapy and/or chemotherapy in testicular cancer. *Cancer*, **42**, 2182.

Seppälä, M. & Ruoslahti, E. (1972) Alpha-fetoprotein in normal and pregnancy sera. *Lancet*, i, 375.

Smithers, D. W. & Wallace, E. N. K. (1962) Radiotherapy in the treatment of patients with seminomas and teratomas of the testicle. *Br. J. Urol.*, **34**, 422.

Storer, G., Vendrik, C. P. J., Struyvengerg, A. & 5 others (1979) Combination chemotherapy with *cis*-diammine dichloroplatinum, vinblastine and bleomycin in advanced testicular non-seminoma. *Lancet*, i, 941.